# Music Listening in Classical Concerts: Theory, Literature Review, and Research Program

**DOI:** 10.3389/fpsyg.2021.638783

**Published:** 2021-04-27

**Authors:** Melanie Wald-Fuhrmann, Hauke Egermann, Anna Czepiel, Katherine O’Neill, Christian Weining, Deborah Meier, Wolfgang Tschacher, Folkert Uhde, Jutta Toelle, Martin Tröndle

**Affiliations:** ^1^Department of Music, Max Planck Institute for Empirical Aesthetics, Frankfurt am Main, Germany; ^2^York Music Psychology Group, University of York, York, United Kingdom; ^3^WÜRTH Chair of Cultural Production, Zeppelin University, Friedrichshafen, Germany; ^4^Experimental Psychology Division, University Hospital for Psychiatry and Psychotherapy, University of Bern, Bern, Switzerland; ^5^Radialsystem V, Berlin, Germany; ^6^Department of Applied Musicology, Gustav Mahler Private University for Music, Klagenfurt, Austria

**Keywords:** concert, music listening, classical music, performance, aesthetic experience

## Abstract

Performing and listening to music occurs in specific situations, requiring specific media. Empirical research on music listening and appreciation, however, tends to overlook the effects these situations and media may have on the listening experience. This article uses the sociological concept of the frame to develop a theory of an aesthetic experience with music as the result of encountering sound/music in the context of a specific situation. By presenting a transdisciplinary sub-field of empirical (concert) studies, we unfold this theory for one such frame: the classical concert. After sketching out the underlying theoretical framework, a selective literature review is conducted to look for evidence on the general plausibility of the single elements of this emerging theory and to identify desiderata. We refer to common criticisms of the standard classical concert, and how new concert formats try to overcome alleged shortcomings and detrimental effects. Finally, an empirical research program is proposed, in which frames and frame components are experimentally manipulated and compared to establish their respective affordances and effects on the musical experience. Such a research program will provide empirical evidence to tackle a question that is still open to debate, i.e., whether the diversified world of modern-day music listening formats also holds a place for the classical concert – and if so, for what kind of classical concert.

## Introduction

Humans love music. We see it as a fitting accompaniment to virtually every situation – using it for a plethora of purposes – and an activity that we engage in daily (Merriam, 1964; DeNora, 2000; Schäfer et al., 2013). We have further developed our passion for music since the invention of music recording, broadcasting, and playback techniques; and even more so since music has become portable and digital and thus all-available (O’Hara and Brown, 2006; Gopinath and Stanyek, 2014). At present, in what the economist and social theorist Jacques Attali (1977, 1985) called “the period of musical repetition,” people in those countries that account for the vast majority of the global recorded music market listen to recorded music about 18 hours a week (IFPI, 2019). Before this, namely throughout the longest part of its history, music could only be listened to when played live. In other words, musicians and listeners had to be co-present, with production and reception occuring simultaneously in situations such as church services or opera theaters, during public festivities, banquets or dance entertainments, and, starting in the late 17th century, in concerts devoted exclusively to attentive music listening (Schwab, 1971; Salmen, 1988; Johnson, 1995; Weber, 1997; Müller, 2014).

Nowadays, live music performance is only one of many ways of listening to and utilizing music and it has to compete with mediatized formats, very similar to other genres of live performances (Heister, 1983; Bontinck, 1999; Auslander, 2008). One could even wonder if homo economicus still needs the concert at all, given the numerous practical and financial advantages of recorded and streamed music. Economically, however, the live music market has not yet fallen behind. In 2019, it was neck and neck with the market for recorded music, either industry creating a global revenue of around 28 billion $ (IFPI, 2020; Statista, 2021). Yet, mostly thanks to music streaming, the market for recorded music could boast an annual growth rate of around 8% per year since 2015, while the live music business has been growing by only about 3% (the COVID-19 pandemic not yet factored in).

While one could leave this for the consumer to decide, publicly funded and subsidized forms of live music, as well as the institutions that have been developed to ensure the public provision with high-quality music performances, face the pressure to substantiate their viability, in addition to their aesthetic and societal relevance. In particular, Western classical music concerts have been challenged. Critics point to shrinking audience numbers, their rapid aging (Heinen, 2013; Gembris and Menze, 2020) as well as the narrow social strata that attend those concerts at all (Reuband, 2007, 2013, 2018). Music managers, orchestras and music festivals are busy with attempts to respond to these calls, giving the classical concert a makeover and restoring its appeal to contemporary and more diverse audiences (Schröder, 2014; Tröndle, 2020). At the same time, the unique character of liveness has found passionate advocates who write about it from an artistic or theoretical standpoint (Gracyk, 1997; Tröndle, 2011, 2018). People are still queuing to listen to famous orchestras, conductors or musicians. New representative concert halls are being built and meeting with enormous public interest, and music festivals are mushrooming in many parts of the world.

The question of whether the diversified world of contemporary music listening formats also holds a place for (different kinds of) classical concerts is still open to debate. At its core stands, we argue, the question whether the concert offers particular and meaningful experiences to its audiences that are qualitatively distinct from those afforded by other musical media (Burland and Pitts, 2014). This is ultimately an empirical question that researchers of liveness in general as well as researchers of the concert and its audiences in particular have only recently started to pursue systematically.

With this article, we want to bring the question of what a classical concert has to offer contemporary audiences to the fore. We present a transdisciplinary sub-field of empirical concert studies with which we expand on earlier ideas of “concert studies” (Tröndle, 2018, 2020) and take up Eric Clarke’s claim for an “ecological approach” to understanding music listening (Clarke, 2005). We start by sketching out the underlying theoretical framework (part 2). From this, we conduct a selective literature review evaluating evidence on the general plausibility of the single elements of this emerging theory and point to desiderata. Along the way, we refer to common criticisms of the standard classical concert and report how new concert formats try to overcome alleged shortcomings and detrimental effects (part 3). Finally, we suggest an empirical research program, in which frames and frame components are experimentally manipulated and compared to establish their respective affordances and effects on the musical experience (part 4).

## Theoretical Core Concepts: Frame, Aesthetic Experience, Classical Concert

### Frame

**FIGURE 1:**
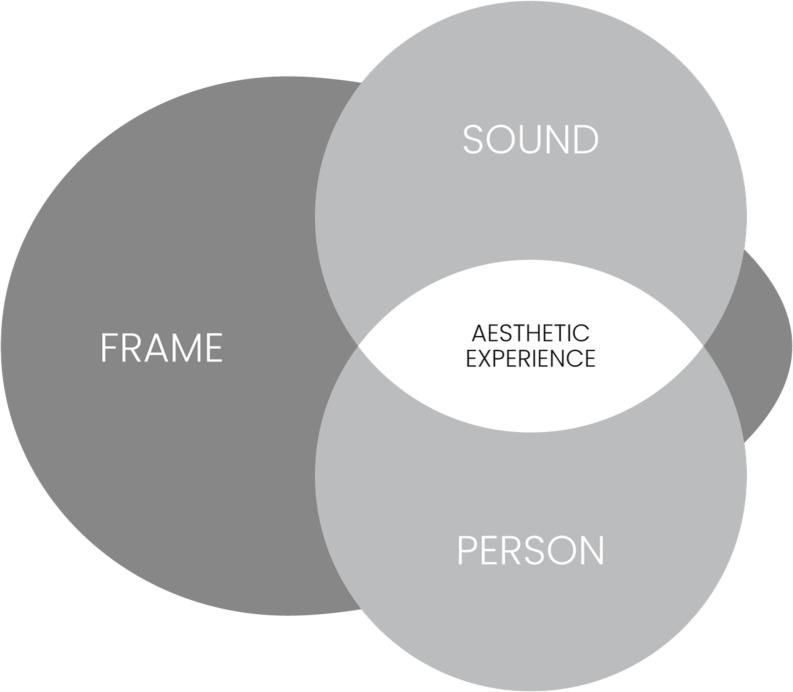
Schematic depiction of a general framework understanding the aesthetic experience of music as the result of the encounter of a person with a sound sequence in a specific frame. Overlaps of Frame and Sound and Frame and Person indicate mediating effects, overlaps of all three components indicate moderating effects of the Frame.

Our approach towards the study of music listening in classical concerts is grounded on a theoretical framework that understands a musical experience as the result of a person’s interaction with a musical stimulus in a specific situation (see Figure 1). A situation encompasses material, social, spatio-temporal, and cultural characteristics. Adopting a term from the sociologist Goffman(1974; see also Willems, 1997), aspects of situations that have a bearing on music listening can be conceptualized as frames, that is, features perceived as essentially belonging to the situation and used by participants to understand and interpret it as well as to align their behavior accordingly. As such, the concept of frame is much more specific whilst simultaneously broader than that of context; a term which is typically used if researchers want to address factors that neither belong to the aesthetic object nor the individual (North and Hargreaves, 2010; Brattico et al., 2013; Leder and Nadal, 2014)^1^. Frames for music listening can be places (e.g., living rooms, cars, concert halls, and public areas), situations (e.g., commuting to work, a romantic dinner, a church service, being alone, or with others), media (e.g., live, recording, digital stream), and discursive contexts (such as a culture’s overarching art and music concepts, or the aesthetics of specific musical styles and genres), all of which are socio-culturally determined.

The frame concept can be related to the theory of embedded cognition. By coining the term “affordance,” Gibson(1966; see also Lewin, 1936) developed a theory of how an object or an environment (implicitly) affects and structures human behavior by virtue of its material and formal properties. Recently, affordances have also been proposed to shape and organize mental processes (Bruineberg and Rietveld, 2014). In the context of music, frames can thus be understood as environmental properties that affect music-related behavior, as well as the mental processes underlying musical experience. In particular, frames in which music is embedded suggest specific listening modes (e.g., attentive, non-attentive, analytical, and emotional), listening behaviors (e.g., sitting still vs. gesturing or dancing), or functions attributed to the music (e.g., for its own sake or aesthetic pleasure vs. mood management, atmosphere creation, or social bonding).

Frames provide a horizon for evaluation and understanding; they can even define if a sound sequence is heard as music at all (e.g., in the case of noise music, it is more likely that it will be heard as music if presented in a typical music frame like a CD or a concert, rather than when heard on the street). The affordances activated by frames are tied to the material properties of the situation (such as space, technologies), as well as the sociocultural meaning attached to them (e.g., concert halls and opera houses as “temples” of high-quality art music performances). Therefore, it can be expected that such frames affect the music experience in bottom-up and top-down ways and act as moderator and mediator variables.

### Aesthetic Experience

The aesthetic experience is a central concept of philosophical aesthetics (Dewey, 1934; Beardsley, 1958; Bubner, 1980; Shusterman, 1997; Küpper and Menke, 2003; Reicher, 2005; Caroll, 2012; Deines et al., 2013). It has also been much studied in psychological aesthetics, where it is discussed also under a variety of other terms such as aesthetic appreciation, appraisal, enjoyment, engagement, perception and evaluation, responses, or, simply, reading, watching, and listening (Abeles and Chung, 1996; Leder et al., 2004; North and Hargreaves, 2005; Marković, 2012; Brattico et al., 2013; Leder and Nadal, 2014; Pelowski et al., 2016). Philosophical concepts and psychological operationalizations, however, do not yet fit together very well. Philosophical concepts emphasize the perceivers’ contemplative, even disinterested attitude (Stolnitz, 1960; Bullough, 1995), their attempt at understanding a piece of art formally and conceptually, as well as the piece’s potential to provide them with a transformative experience. In contrast, psychological theory would conceptualize aesthetic experience as an output variable in the context of a stimulus-response model, with outputs such as liking, aesthetic judgment and elicited emotion, and their physiological and neurological correlates. Recently, more specific qualities of aesthetic experiences were proposed, i.e., aesthetic emotion (Marković, 2012; Juslin, 2013; Schindler et al., 2017; Menninghaus et al., 2019), fascination (Marković, 2012), awe (Konečni, 2005), or being moved (Konečni, 2005; Menninghaus et al., 2015). In general, however, psychological research still emphasizes a primarily passive, physical, and emotional understanding of aesthetic experience, whereas philosophical theorizing tends to apply an overly cognitivist concept.

In psychology and philosophy of mind, a lived experience is generally defined as a first-person, qualitative phenomenon (Chalmers, 1995). Experiences are distinguished from objective response phenomena, such as physiological and behavioral processes. Experiences have qualitative properties (“qualia”), and they are elements of cognitive and emotional processes. In the terminology of phenomenology, qualia comprise “what it feels like” to have exactly this experience in the here-and-now (Nagel, 1974). Cognition refers to the processing of information through mental representations, thought, evaluation, the activation of memory traces and schemata. Cognition can, but need not, be conscious and experienced, sometimes even in a linguistic form as inner speech. Lastly, emotions are generally experienced. Emotions lend a specific flavor to experiences, thus the experience of joy, sadness, fear, or any number of further emotions or mixtures of emotions.

For the remainder of this paper, we will continue with a provisional comprehensive concept of an aesthetic experience of music that combines facets of existing philosophical, aesthetic, and psychological concepts. We conceive of it as a person’s phenomenal state while attending to and internally interacting with a sequence of sounds primarily for the sake of its perceptual and formal properties and their possible meaning, but not so much its real-life information value. In the case of a temporally unfolding stimulus as music, such a state is necessarily dynamic and may combine feelings, perceptions, emotions, associations, expectations, and insights, as well as the evaluation of the musical piece itself and the state(s) into which it puts the listener – all of them mutually influencing each other. It is related to a listener’s present attitude and degree of attention, and comes with physiological, motivational, and behavioral responses.

### The Classical Concert as a Frame for Music Listening

This paper claims that a classical concert is one particular frame for music listening, which shapes the aesthetic experience of the music featured within it in specific ways, and that we need empirical studies to test this claim and understand the underlying mechanisms. But which of its characteristics are most likely to guide and influence the experience of a piece of music? Existing descriptions and theories, as well as results that have emerged from qualitative empirical studies (Heister, 1983; Gracyk, 1997; Small, 1998; Auslander, 2008; Gross, 2013; Burland and Pitts, 2014; Tröndle, 2020), point to two defining factors of the classical concert frame: its work-centered aesthetics and its liveness.

As a result of the co-evolution of its forms, its discourses, and its repertoires, the concert has developed into the embodiment (and driving factor) of a specific and presupposition-rich musical aesthetics (Johnson, 1995; Weber, 1997; Müller, 2014; Tröndle, 2020). Heister, who has provided the most exhaustive theoretical concept of the classical concert so far, defines it as the “place where musical autonomy is realized” (Heister, 1983, p. 42). A concert, at least in the form it has taken on in the late 19th and early 20th century, publicly celebrates the idea of the musical artwork, which is literally placed centerstage (Goehr, 1994). The musicians have to devote all their skill and artistic refinement into the work’s realization. Meanwhile the audience, which first had to learn “the art of listening” (Gay, 1984), has to receive it with concentration, even contemplation, and reverence, in an act of “purely aesthetic and musical savoring” (Heister, 1983, p. 522ss).

Almost all other characteristics of the concert are direct consequences of this aesthetics, as Heister meticulously spelled out. On the one hand, concert hall acoustics, program selections, the training of professional musicians, and the behavioral regimes of sitting still and quietly seek to provide optimal conditions for the production and reception of the greatest musical works (Gross, 2013). On the other hand, the building and design of concert halls, a certain cult of great names and charismatic artists – be they composers or performers – formal dress codes, and rituals serve as constant reminders of the ideology of autonomous music (Cressman, 2012).

The other main factor of a concert is its nature as a live performance featuring the distinct, but interrelated roles, of performers and listeners. This spatio-temporal co-presence entails a number of other aspects, most importantly the possibility to watch the performers creating the music and the genuinely social and interactive character of the event (Gracyk, 1997). Although the concert has typically been seen as the pure embodiment of presentational performance (Besseler, 1926, 1959; Turino, 2008), recently, social-interactive and participatory aspects have been identified as well. As a live performance, a concert affords (verbal) communication between audience members (at least before the concert and in the pause of classical music concerts), inviting participants to form a short-lived community (Cochrane, 2009; Burland and Pitts, 2014). It can also lead to manifold interaction processes: audience members can show support, interest, attention and appreciation, or displeasure, thus providing feedback to the performers which they are then likely to respond to, closing the autopoietic feedback loop (Fischer-Lichte, 2004).

Apart from its social character, liveness is also typically associated with ideas of immediacy, indeterminacy, uniqueness, and non-repeatability of the event (Auslander, 2008). Neither the audience members nor the musicians know exactly how the performance will turn out, which might be seen as another mechanism of directing and fixing the audience’s attention. This, in turn, lends presence and an event-like character to a performance, which comes with the promise of a not only quantitatively, but also qualitatively unique experience – a feature of present-day leisure culture that is very much sought-after by audiences (Schulze, 1992; Gumbrecht, 2004a; Seel, 2008; Tröndle, 2011; Rebstock, 2020).

In sum, the concert is a frame for music listening that is supposed to provide optimal conditions for the purely aesthetic contemplation of (excellent performances of) great musical works together with like-minded people. This historically evolved frame might afford a specific concert experience which consists of a certain type of listening (being pleasurably immersed into the music), a multi-modal character of the stimulus, its social embeddedness (feeling as part of a community), and the appreciation of its singular character. Such experiences have been described in qualitative studies and claimed by theoreticians and advocates of the genre (Heister, 1983; Radbourne et al., 2014; Rebstock, 2020), but not yet quantitatively corroborated. In addition, while the standard form incorporates implicit assumptions about the relationship between its features and the hoped-for experience resulting from them, new and experimental concert formats that have been developed over the past decades can be understood as a form of aesthetic and social critique of the standard format (Brüstle, 2013; Schröder, 2014; Roselt, 2020). Typically, these new forms modify the venue, the forms of listening, but also the relationships between performers and audience members and their respective rituals. By singling out and modifying such elements, they point to their potentially detrimental effect on the aesthetic experience and at the same time exemplify how this could be overcome to allow for fresh, heightened and new musical and social experiences that can also have the potential to attract younger audiences or audiences from other social and cultural backgrounds. Thus, they also tend to shift the focus of a concert away from the musical work toward the event-like aspects of a live performance.

This apparent conflict between existing concert formats points to the gap between implicit assumptions of concert practitioners and the lack of empirical knowledge about how exactly the elements of a concert – individually, as well as jointly – contribute to listeners’ actual experiences. Further, each element can, in principle, be realized in a multitude of ways, which might in turn substantially affect the degree and direction of its effect. This as well has not been examined empirically.

## What We Already Know About Music Listening in Classical Concerts, and What We Still Need to Know: A Literature Review

To date, concert research consists of several branches. Of these, the history of the concert, its repertoires, halls, and listening forms (Schwab, 1971; Heister, 1983; Forsyth, 1985; Salmen, 1988; Johnson, 1995; Weber, 1997; Cressman, 2012; Thorau and Ziemer, 2019), as well as the demography, sociology and consumer behavior of audiences (Dollase, 1998; Reuband, 2007, 2013; Glogner-Pilz and Föhl, 2011; Gembris and Menze, 2020; Tröndle, 2020) have been examined most comprehensively. More recently, a number of qualitative studies has addressed also the motivations and experiences of various audiences (Pitts, 2005; Roose, 2008; Dobson, 2010; Gross, 2013; Brown and Knox, 2017; Toelle and Sloboda, 2019). There are studies which adopted a quantitative approach in measuring listeners’ experiences in concerts, by collecting continuous or retrospective self-report data or physiological recordings (McAdams et al., 2004; Thompson, 2006; Egermann et al., 2013; Stevens et al., 2014).

Although the specificity of the concert as a medium or format for music listening has theoretically been identified sufficiently well, musical audience research has not yet addressed this issue systematically. Typically, audience experiences are neither analyzed with regard to which of their components are concert-specific, nor are frame effects explicitly addressed. This is related to the fact that music psychological research in general tends to overlook situational and frame effects (Clarke, 2005). Even if studies had been conducted during live concerts, this context was so far neither explicitly addressed nor experimentally manipulated. Likewise, if concerts were compared with other musical media, the focus was not on actual experiences but listening times (Roose and Vander Stichele, 2010).

This is very different from the situation in museum studies, which were the first to experimentally address the effects a museum, and the way it displays and communicates artworks, has on the experience of visitors (Falk and Dierking, 1992; Stuffmann, 2005; Brieber et al., 2014).

In the following sections of this chapter, we come back to the most distinctive features of a concert identified above and point out what they might contribute to the afforded musical experience. We summarize related results from the fields of concert and audience research. We also identify desiderata and refer to other research contexts and approaches that might prove fruitful for the endeavor of understanding how concerts frame and affect music experiences.

### Effects of Venue

Concert halls, the majority of which have been built since the 19th century, are both a prerequisite for a performance-centered staging of classical music and a potent sign of the concert’s underlying aesthetics. By their mere existence as buildings specifically dedicated to hosting musical performances, they signal an assumed importance, seriousness, and high-art quality of the music and the entire event of going to a concert. The architectural style and design of the hall is an aesthetic stimulus in itself that creates a specific atmosphere. Further, their acoustics co-constitute the auditory musical stimulus.

A concert hall is also perhaps the most influential component in the concert regime, as it materially affords what people can perceive and do within such as situation: the tiers require everyone to sit during the performance. Their spatial arrangement directs the audience’s attention to the stage by orienting them physically toward it. Although usually, parts of the audience can also be seen, the lighting control makes it clear that this is only accidental and that the audience should focus on the performers onstage. Taken together, the effect of a concert hall on the musical experience can be studied with regard to (1) the atmosphere created, (2) its function as a framing and/or priming intervention, (3) its contribution to the actual acoustic stimulus, and (4) the behavior it affords.

(1) The concept of atmospheres stems from phenomenology and has engendered broad and mostly theoretical research in the past years with strong affinities to aesthetic contexts and questions (Böhme, 1995, 2006; Griffero, 2014; Schmitz, 2014). It refers to the perception and experience a certain (often architecturally defined) space affords, but also to the social interaction that takes place in that space and theorizes upon the effects a certain atmosphere has on the experience and behavior of an individual. Psychological studies on the perception of atmospheres still are a desideratum (Schönhammer, 2014; but see Tröndle and Tschacher, 2012 for a first example), although practitioners in the fields of concert hall architecture and concert locations are aware of this issue (Göbel, 2020; Kirchberg, 2020). Today, concert series or festivals in particular, as well as individual concerts, are often staged in unusual locations. Such locations comprise, among others, of castles, museums, churches, factories, farms, outdoor stages, or dance clubs. In the case of the Yellow Lounge concert series in Berlin, its organizers from Deutsche Grammophon advertise it with particular reference to an altered atmosphere: “classical music can thrill even outside of the concert hall, good-humored and fully relaxed in the Club. (…) Good drinks, communicative atmosphere^2^.” Qualitative research has provided first evidence that festival audiences take note of and appreciate specific atmospheres and see them as a factor that positively influences their experiences (Karlsen, 2014). However, no research so far has examined how exactly the experience of one and the same piece of music differs when listened to in a barn as compared to a hall in a palace, or in a concert hall with modern architecture as compared to one in the styles of the 19th century.
(2) A large body of market, media, and social psychology research shows that people’s judgments, interpretations, and experiences of any given phenomenon can be modified by priming or framing. While priming is conceptualized as additional information that influences the appreciation of a subsequent stimulus, framing means to select and highlight specific aspects inherent to a stimulus in order to modify its appreciation (Entman, 1993). Emotionally charged framing information (Entman, 1993), as well as those implying a positive or negative evaluation (Levin et al., 1998) have been found to be particularly powerful. The latter is also related to the so-called prestige effect.
  That these effects also work in the contexts of the arts in general (Tröndle et al., 2014; Tröndle and Tschacher, 2016), and in music has been shown by a number of studies (for a recent overview, see Fischinger et al., 2020). Effects of program notes and other additional information in the form of texts or images have been found for emotions induced by music (Vuoskoski and Eerola, 2015), enjoyment of music (Margulis, 2010), children’s attention and comprehension (Margulis et al., 2015), evaluation (Anglada-Tort and Müllensiefen, 2017; Fischinger et al., 2020), and even perception of basic musical characteristics (Chapman, 1981; North and Hargreaves, 2010; Fischinger et al., 2020).
  That listeners might wish for additional information helping them understand and appreciate a piece of music is plausible. Most people lack advanced musical training to be confident in their judgment about any work and performance. In addition, the meaning of musical elements is typically far from being clear but contains a large degree of ambiguity. Even more, in the context of the arts, there simply are no such things as objective value and meaning, according to Umberto Eco’s theory of the open work (Eco, 1989).
  So far, priming and framing information about music have been studied in the form of texts or images. These media also play a role in the context of a concert, be it in the form of programs, advertisements, paintings or sculptures of famous composers, program notes, or introductory talks. However, the atmosphere of a concert hall has not yet been researched with regards to its potential nature as prime and frame.
(3) A concert hall provides a specific acoustic setting ideally optimized for performances of classical music (Lindau, 2010). Tajadura-Jiménez et al. (2010) were among the first to test the effect of room size and sound direction on emotional responses to natural and artificial sounds. They observed that sound sources in front of listeners were perceived as less arousing than those behind listeners, while the sound of a large concert hall was experienced as more arousing and negatively valenced than the sound of a small room. This finding was explained by the additional observation that the larger room in the experiment was also perceived as “less safe” than the small room. Furthermore, Pätynen and Lokki (2016) showed that concert halls with a traditional rectangular shape evoke stronger physiological (skin conductance) and subjective responses to music presented in them (in this case excerpts from Beethoven’s Seventh Symphony). In sum, however, empirical research that compares perception of – and responses to – acoustical variations of the same musical pieces is scarce.

(4) The behavioral restrictions created by the design of the auditorium together with learned norms are meant to provide, on the one hand, the condition for an undisturbed, attentive, even immersive listening experience in a specific time-frame. On the other hand, such restrictions also favor disembodied listening and the suppression of any overt spontaneous response. Originally a necessity to make music audible to a large group of people, this aspect of a concert has been met with the sharpest criticism in a time in which undisturbed listening is always possible via radio and recordings. In particular, the discouragement of overt and spontaneous interaction between participants might be experienced as antagonizing the inherently social nature of a concert (Small, 1998). The implicit, but nonetheless perceivable, behavioral norms can produce stress in first-time and only occasional attenders thus preventing them from attending at all (Radbourne et al., 2009; Dobson, 2010; Dobson and Pitts, 2011; Tröndle, 2019). The focus on contemplative and disembodied listening might counteract bodily entrainment afforded by some pieces. Some concert organizers have started to address this criticism by experimenting with concert formats that allow the audience to behave differently, e.g., lying down or walking around instead of sitting still, or by providing opportunities for real interaction and spontaneity.
  A significant strand of research supports the concept of embodiment among various disciplines (e.g., developmental, social, and clinical psychology). Especially in the field of music psychology, researchers demonstrated the impact of bodily responses on listeners’ music experiences and vice versa. Particularly strong is the urge to move elicited by rhythmically accentuated music with a salient beat, which has been discussed as sensorimotor coupling (Janata et al., 2012; Stupacher et al., 2016).
  Although a considerable amount of research has already been conducted and published around embodied music experience, literature that examines effects of listening contexts, media, and frames is scarce. As a first step, it has been shown that participants’ non-verbal responses to live music differ from those to recorded music. For example, Swarbrick et al. (2019) found that head movements were faster during a live performance of a Rock musician than during the recorded version as well as finding that movements of self-identified fans being faster and having higher degrees of rhythmic entrainment (movement to beat) compared neutral listeners.
  Further, research on non-verbal behavioral synchrony – which refers to the temporal coupling of movement or physiology between at least two individuals – is closely linked to the concept of embodiment and is viable in social listening situations. Although non-verbal synchrony research in the classical concert is still in an early phase, studies in realistic concert settings have so far revealed significant non-verbal synchrony effects within the audience. For example, Seibert et al. (2019) examined the spontaneous coordination of bodily movements – dyadic temporal coupling – within audience members and between audience members and musicians in a classical (chamber music) concert. They found strong movement synchrony between musicians, and also small to medium movement synchrony within the audience, despite the behavioral norms of sitting still. Aspects of music experience, namely absorption and the feeling of being connected to the musicians, were significantly negatively associated with non-verbal synchrony.
  In addition to movement synchrony, some studies have explored physiological synchrony across audience members as an index of an embodied experience. Sato et al. (2017) investigated respiratory activity and emotional states within fifteen audience members of a live concert, where they found that respiratory synchronization effects emerged from time to time. Importantly, participants’ excitement seemed to correspond with the respiration activity indicated by synchronized respiratory phases. In another concert study, Bernardi et al. (2017) found that cardiorespiratory synchrony among audience members were higher during live music listening, compared to a resting baseline. In corroborating findings of Sato et al. (2017), Bernardi and colleagues also found that synchrony and ratings of pleasantness were positively correlated; though it should be noted that synchrony was more strongly correlated (i.e., more variance explained) with low-level acoustic features such as loudness variability (compared to pleasantness ratings). Thus, it could be argued that the quality of performance in terms of excitement and pleasantness can be estimated – at least to a certain extent – by synchronous phase respiration.
  Accordingly, the presented results on non-verbal synchrony and its association with perceived quality of performance and music experience underlined the embodiment perspective and stresses the relevance of embodied musical experience despite behavioral regimes that try to suppress it.

### Effects of Multi-Modal Perception

As a consequence of the co-presence of performers and audience, music in a concert becomes a richer, multi-modal stimulus. In particular, visual aspects might add layers of meaning and aesthetic affordance to the musical sound. Studies and deliberations of a more general kind have argued that aesthetic pleasure is most commonly evoked by combining multi-modal perception into one single experience, including sight, sound, environment, and company (Cohen, 2009; Huron, 2012). So far, potential multimodality effects in music listening and concerts have been primarily studied with regard to visual aspects, i.e., (1) visual aspects of the concert hall and performer, and (2) performers’ gestures. But it can be assumed that aspects of vibrotactile perception of sounds, room temperature and climate, lighting, or seating comfort might also affect the experience within a concert.

(1) The style and design of a concert venue provides a very strong visual stimulus, which may affect audience members’ emotions and level of engagement and to which they will respond with a judgment of taste (Cook, 2012). By investigating the concert setting, a study by Coutinho and Scherer (2017) compared emotions during a live performance in a real-world musical context in a church (as part of a Lieder recital) to the audio-video recording in a laboratory situation (university lecture hall). Self-reports of emotion engagement, feelings of wonder and tenderness were much higher in the church setting, while boredom, tension and sadness were higher in the lecture hall setting, showing that environment could indeed be a crucial component in evoking more intense aesthetic emotions. Equally, fashion is a field where visual properties carry meaning and where human tastes vary a lot (Solomon, 1985). In a concert, it is present via performers’ attire. For example, formal dress can create a “sense of occasion” (Griffiths, 2011) and increase the perception of a performer’s technical and musical proficiency (Griffiths, 2010).
(2) How auditory information interacts with performer gesture has been widely examined in psychology, specifically in the field of multi-modal perception. Such gestures can provide additional information about the music’s expressive and structural properties, thus enabling the audience to enter into a more engaging internal dialog with the musical pieces. For example, it has been shown that musical expertise can be perceived through performer movement, even in the absence of any auditory information pointing to a substantial effect of performers’ movements and gestures (Tsay, 2013; Griffiths and Reay, 2018). Additionally, performative and expressive movements of instrumentalists (Davidson, 1993, 2012; Broughton and Stevens, 2009; Vines et al., 2011; Silveira, 2014; Vuoskoski et al., 2014), singers (Davidson, 2001; Lange et al. in review), and conductors (Luck et al., 2010; Morrison and Selvey, 2014), show that gestures can increase perceived expressivity of the music. Movements of a performer can further enhance communication of tension (Vines et al., 2004) and emotion of the music (Dahl and Friberg, 2004), as well as the emotion of the performer (Van Zijl and Luck, 2013), to an audience.
  Using psychophysiology as a measure of felt affect, Chapados and Levitin (2008) demonstrated that electrodermal activity (representing felt arousal) was significantly higher in audiovisual performances of Stravinsky’s Second Piece for solo clarinet, compared to audio-only and visual-only performances. Together with evidence showing that performer movement increases perception of expressivity, emotionality, and skill, this suggests that the visual component of a live concert performance can enhance our experience of the music. Indeed, first-time concertgoers commented on how they felt the visual cues enhanced enjoyment of the music (Dobson, 2010; Dobson and Pitts, 2011).

However, there is also some research showing that visuals do not seem to enhance the emotional experience in listeners. Finnäs (1992) found no significant difference of subjectively rated felt emotional response (of either musicians or non-musicians) between audio-only and audiovisual versions of Mahler’s Second Symphony. Vuoskoski et al. (2016) found that audio-only performances of Brahms’ piano Intermezzo in B minor – compared to audiovisual performances – elicited more emotional arousal (as indexed by skin conductance), contrary to findings of Chapados and Levitin (2008). The authors discuss how musical styles (Romantic vs. Modern) and the degrees of freedom of the performer (a clarinetist who is standing up compared to a pianist who is sitting down) may influence the extent to which visuals play a role in musical experience. Thus, the specific role of visuals as an enhancer in live music experience still requires further empirical study to consider possible variables (styles, instrument, and musical expertise of perceiver), as well as considering these factors in more applied and multi-modal contexts, such as a concert setting.

### Effects of the Social Character of Music Listening

The presence and visibility of musicians, as well as the group nature of the audience, lend a social, and participatory component to the aesthetic experience. This social component is moderated, however, by behavioral protocols, arrangement of tiers vs. stage, and existing power relationships.

Qualitative research shows how much listeners appreciate the social nature of a concert and whether it is able to induce feelings of a shared experience with peers, a sense of belonging, direct interaction with the performers, and participation in something meaningful (Radbourne et al., 2014). The possibility to watch performers is often mentioned as a positively experienced element of concerts alongside a real interest in personal connections with performers (Burland and Pitts, 2014).

Quantitative and experimental studies have further corroborated these qualitative findings, in particular regarding the social character of the audience. For example, it was experimentally demonstrated that social feedback about other music listeners’ enjoyment changes how listeners respond to music subjectively, where knowledge of previous ratings of a musical performance influences an individual, motivated by a desire to conform (Egermann et al., 2013). This finding was interpreted as a form of normative social influence on social appraisals (Manstead and Fischer, 2001) assuming that a similar mechanism could be activated in classical music concerts through social feedback via (*inter alia*) applause (Mann et al., 2013).

Previous research has demonstrated the effect the presence of other people has on a listener’s response to music. The emotion experienced when listening to music, specifically strong experiences with music, has been shown to be influenced by the social context in which the listening occurred (Gabrielsson and Wik, 2003), with intense experiences occurring more frequently in live concerts when other people were present (Lamont, 2011). In a more controlled study that utilized recorded stimuli – where participants listened to self-chosen or randomly sampled music samples – more intense emotions were reported when participants were listening with a close friend or partner compared to when listening alone (Liljeström et al., 2013). However, another study found that listening in a group does not lead to more intense emotional responses perhaps due to less concentration on the music (Egermann et al., 2011). In a later study by Linnemann et al. (2016), music was found to reduce stress more if it was listened to in the presence of others, regardless of the original motivation for listening to the music, where influence of others has been found to be stronger if they are known to the listener.

Research on the effects of an interaction between listeners and performers, however, is much more sparse. Here, qualitative studies also provide evidence for the general importance and appreciation audiences and performers attribute to it (Moelants et al., 2012; Toelle and Sloboda, 2019). In the behaviorally restricted setting of a classical concert, however, real and spontaneous interaction is only possible to a small degree. The only legitimate form of mutual feedback is applause (Toelle, 2018), which not only informs the musicians how the audience is responding to their performances, but also provides feedback to an audience member on the reception of the music by other audience members.

If compared with other concert types, such as jazz and popular music concerts, classical concerts seem to leave the potential of creating a social experience of music largely unused, which is one factor behind the different experiences these concert types can afford (Pitts, 2005; Kulczynski et al., 2016). This has not only been criticized from a theoretical point of view (Small, 1998), but also been addressed by performers and concert curators who have started to experiment with forms that invite true interaction and even participation (Schröder, 2014; Toelle and Sloboda, 2019). So far, the underlying assumptions as to how exactly such changes impact the collective experience have not yet been explored in a systematic way. The need to test these assumptions in a multi-disciplinary and ecologically valid way is central to further the understanding of the social experience of a concert and how the group experience can be enhanced.

### Effects of Presence, Uniqueness, Immediacy

The live character of a concert is also closely tied to its nature as a single, unique, and un-repeatable event that might be valued for its presence and immediacy (Auslander, 2008). According to Walter Benjamin, this special quality of a live concert could be described as the “aura,” i.e., the authenticity, realness and presence of the aesthetic object that technical reproduction would not be able to recreate (Benjamin, 1963). Gumbrecht (2004b) even puts “presence” in the focus of the aesthetic experience as the sensorial and lived experience of an appearance. Further developing this concept of presence in the context of classical music concerts, Rebstock (2020) claims that the production of presence might be the key component of a concert and, therefore, new concert formats should aim for a higher intensity of presence (Rebstock, 2020).

Although the concept of presence in the context of aesthetic experiences is repeatedly discussed in theory (Seel, 2003; Fischer-Lichte, 2004; Gumbrecht, 2004b; Rebstock, 2020; Roselt, 2020), no specific empirical research on it seems to exist, supposedly because of its intangible nature. However, some of the studies mentioned in the above sections include certain components, as the experience of presence can be understood as a sensorial and intense physical experience and is *per se* part of the aesthetic experience (Gumbrecht, 2003).

While on the one hand the “incursion of reproduction into the live event” (Auslander, 2008) can be seen as a threatening development for the live experience, on the other hand it can be argued that technical reproduction might increase the demand of experiences of unmediated presence or is already even a fixed component of auratic moments (Schulze, 2011). Either way, there is no denying the fact that the link between uniqueness and presence dissolves due to technical developments, which empirical research might take up in a fruitful way.

## Toward a Research Program

Our literature review has shown that the (classical) concert and concert listening experiences have been already acknowledged as worthwhile research topics by a multitude of disciplines. At the same time, more thorough, systematic, and transdisciplinary research is still needed since (so far) a theoretical framework able to generate interrelated research questions and overarching hypotheses has not been postulated. In the final passages of our paper, we develop what we have outlined in the preceding sections into a sketch of a research program that, albeit functioning within a psychological scheme, is genuinely interdisciplinary. In a nutshell, this research program stipulates the comparison and experimental manipulation of frames as well as frame components to establish their respective affordances and effects on the musical experience. Here, the aesthetic experience of music is the dependent variable of interest. Although it is brought about by listening to a specific performance of music, i.e., the stimulus, and how a listener interacts with it, this is not what will be of primary interest. Rather, the focus will lie on the mediating and moderating effects of the concert frame, as well as its interactions with person- and stimulus-related factors.

### Frame Components as Stimuli

**FIGURE 2:**
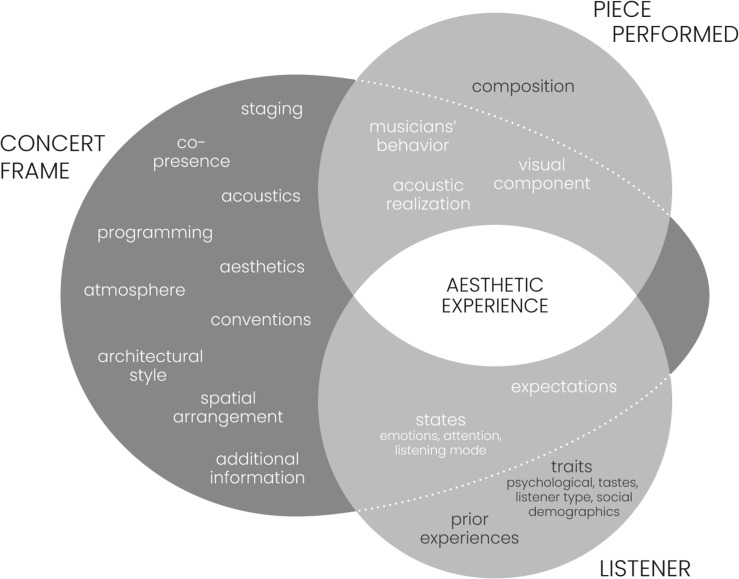
Expanded frame-music-listener model to show potential concrete mediating and moderating effects of concert components on the performance and the listener and thus, the aesthetic experience of music.

At the heart of empirical concert research, as we propose in this current article, stands the idea of using frames and their individual components as stimuli and manipulating them experimentally, in order to establish the nature, form, and strength of their influence on the musical stimulus and the affordances they unfold for performers and listeners (see the expanded model in Figure 2). By nature, we mean whether an effect enhances or disturbs the experience, and on which component(s) of the aesthetic experience it exerts its influence. By form, we mean whether an effect is rather a mediating or moderating one. While some aspects of frames can be studied in the lab (which should be acknowledged as a particular frame of its own), frames that largely rely on particular venues and social situations cannot. This is especially true for the concert. While virtual reality technologies might provide a more lab-like solution for this in the near future, at the moment, researchers have to go to or create such frames and situations themselves.

In essence, series of experiments need to be designed in which individual concert components are manipulated. Inspirations for the components and types of such experimental manipulations should be derived from contemporary (and possibly even historical) concert practices, most importantly from practitioners who critically reflect on the concert (Rebstock, 2020; Roselt, 2020). In such a case, the pieces to be performed as well as the musicians performing them need to remain the same to control for (the largest part of) the acoustic stimulus. Components to be manipulated would be those that have been shown to define the concert as a concert, namely: the venue and the atmosphere it creates, the multi-modality, the listening mode, the behavior, and the social component. This will, at least partially, result in concerts that have a very different character and atmosphere, concerts whose frame function will thus become increasingly more “visible” up to a degree where it might no longer be working as a frame, but as an artistic stimulus in its own right, completely merged with the music.

In terms of the venue, a considerable number of effects on the musical stimulus and the listeners can be studied, that likely work either in the form of mediators or moderators. A manipulation in the form of performing the same program in halls with different acoustics, architectural styles, layout of tiers and stage, and social connotations suggests itself. While the acoustics have an effect on the sound of the performed piece, the atmosphere and architecture may likewise influence the state of the listeners. Potential priming effects of style and decoration of a venue, however, might primarily work as moderators on how a listener experiences the music.

The multimodal character of music in a concert, that follows from the co-presence of performers and listeners, is also a way in which the concert exerts an influence on the musical stimulus or even contributes to it. It can be examined by varying the visual appearance of performers, their behavior toward the audience, the degree and form of their overt interaction with each other, their display of their own emotions and engagement. An extreme form would be to hide performers from sight, as was repeatedly proposed and realized by historical theoreticians and practitioners of the concert since the 17th century (Schwab, 1991; Schröder, 2014). Besides, existing ideas to increase the value of the visual aspect even more by an artistic design of lighting, stage decorations, or the integration of video projections could be taken up and experimentally explored if they have an effect on attention, immersion, understanding, and appreciation.

Although, as a consequence of its underlying aesthetics, the attentive and disembodied listening mode is the historically preferred one in a concert, neither do all concerts afford these to a satisfying degree, nor should other potentially pleasurable and meaningful listening modes be excluded due to ideological reasons. Manipulations of a concert in the attempt to afford a specific listening mode is therefore another potential area of experimental variation of the classical standard concert. Such listening modes could include the exploration of the embodiment of the music, listening emotionally, associatively, or auto-biographically.

Related to this is the aspect of behavioral regimes exerted by a standard concert that can be assumed to moderate listening experiences. Here, moreover, variations can be designed that explore which other (less strict and ritualized) behaviors are possible and how they change the experience of the music.

Further, the social component needs careful consideration. Obvious variations could target the relationship between performers and audience in the attempt to make it less hierarchical, more spontaneous, personal, interactive, and – on the side of the audience – more participatory. Also, moderating effects of the size, density, and spatial arrangement of audiences can be examined.

Finally, variations could address the aspect of a perceived event-character and uniqueness of a concert. Here, elements that enhance the degree of surprise and indeterminacy would be related to the programming, the staging of pieces, or the integration of improvisation, among others, and thus moderate their experience.

All such variations would have to fulfill the double need of making sense artistically as a concert and of singling out individual components. To achieve this, concert curators need to form an essential part of a research team. Any hypothesis underlying a concrete experimental manipulation should primarily regard direct and mediated effects on attention, relating to the music, making sense of it, perceived presence and event-character, and the social components of the concert experience. This is because the distinctive features of a concert can all be seen as meant to afford intense, immersed, unique, and personally meaningful musical experiences that are characterized by two dimensions of relationships: between the individual listener and the music, and between the listeners and performers.

### Perspectives

Such a research program that thoroughly and empirically investigates, as well as manipulates, the concert frame and its components can only be performed in interdisciplinary teams that gather musicologists, sociologists, concert practitioners, and under the guidance of psychologists (Tröndle et al., 2020). It will come with a lot of challenges, above all methodological and conceptual ones in order to balance control with realism. However, it also provides important perspectives and promises to greatly advance (music) psychology, (cultural) sociology, and (empirical) aesthetics. In particular, it places a defining aspect of the art of music centerstage, namely that music requires to be mediated by performers, technologies, and even the air circulating through particular rooms. At the same time, our concept of frame highlights an aspect that is not only relevant, but crucial for all art forms. How (art) objects are perceived and experienced is only in part a direct result of their sensory and formal properties, but depends to a large degree on the aesthetic, social, and cultural discourses creating and surrounding them, as well as the situations in which they are perceived (Clarke, 2005). Artifacts, cultural objects, and art works in particular do not have a meaning of their own, but gain their meaning from cultural practices and discourses in which they are embedded. The concert provides a particularly convenient example to embark on a systematic exploration of effects of frames – their situational, social, multi-modal, and discursive constituents – on one set of aesthetic experience, the experience of music. Thus, we can expect to gather insights that will help us answer the initial questions, whether and in what respects music listening in classical concerts is different from other listening frames, and also, which types of concerts may continue to be of aesthetic interest to contemporary societies.

## Author Contributions

MW-F created a first and second draft of the article, prepared figures, wrote most of the sections, and led the process. KO’N, HE, AC, CW, DM, WT, JT, and MT wrote individual sections of the article. All authors discussed and revised earlier versions of the manuscript and read and approved the final manuscript.

## Conflict of Interest

The authors declare that the research was conducted in the absence of any commercial or financial relationships that could be construed as a potential conflict of interest.

## Footnotes

1 A frame conceptualized as a set of concepts that organize experiences guiding the actions of individuals and groups, can be closely connected to Michael Focault’s idea of the dispositif. Goffman is referring to the experience, Focault to power, both theories stem from the 1970s and try to conceptualize human behavior.

2 http://www.communicating-music.eu/en/345/yellow-lounge-berlin-de.html, 19 June 2020.
